# Patient-Derived Organoids Recapitulate Pathological Intrinsic and Phenotypic Features of Fibrous Dysplasia

**DOI:** 10.3390/cells13090729

**Published:** 2024-04-23

**Authors:** Ha-Young Kim, Clémentine Charton, Jung Hee Shim, So Young Lim, Jinho Kim, Sejoon Lee, Jung Hun Ohn, Baek Kyu Kim, Chan Yeong Heo

**Affiliations:** 1 Interdisciplinary Program in Bioengineering, Seoul National University, Seoul 08826, Republic of Korea; hkim247@snu.ac.kr; 2 Department of Plastic and Reconstructive Surgery, College of Medicine, Seoul National University, Seoul 03080, Republic of Korea; 3 Precision Medicine Center, Future Innovation Research Division, Seoul National University Bundang Hospital, Seongnam 13605, Republic of Korea; chartonclementine@gmail.com (C.C.); jinho80@gmail.com (J.K.); sejoonlee@snubh.org (S.L.); 4 Department of Research Administration Team, Seoul National University Bundang Hospital, Seongnam 13620, Republic of Korea; 98757@snubh.org; 5 Department of Plastic and Reconstructive Surgery, Seoul National University Bundang Hospital, Seongnam 13620, Republic of Korea; plasrecon@snubh.org; 6 Department of Plastic and Reconstructive Surgery, Samsung Medical Center, Sungkyunkwan University School of Medicine, Seoul 06351, Republic of Korea; sy00.lim@samsung.com; 7 Department of Laboratory Medicine, Seoul National University Bundang Hospital, Seoul National University College of Medicine, Seongnam 13620, Republic of Korea; 8 Department of Pathology, Seoul National University Bundang Hospital, Seongnam 13620, Republic of Korea; 9 Department of Internal Medicine, Seoul National University Bundang Hospital, Seongnam 13620, Republic of Korea; 65824@snubh.org

**Keywords:** fibrous dysplasia, rare disease, bone lesion, patient-derived organoid, fibrosis, scRNA sequencing, GNAS mutation

## Abstract

Fibrous dysplasia (FD) is a rare bone disorder characterized by the replacement of normal bone with benign fibro-osseous tissue. Developments in our understanding of the pathophysiology and treatment options are impeded by the lack of suitable research models. In this study, we developed an in vitro organotypic model capable of recapitulating key intrinsic and phenotypic properties of FD. Initially, transcriptomic profiling of individual cells isolated from patient lesional tissues unveiled intralesional molecular and cellular heterogeneity. Leveraging these insights, we established patient-derived organoids (PDOs) using primary cells obtained from patient FD lesions. Evaluation of PDOs demonstrated preservation of fibrosis-associated constituent cell types and transcriptional signatures observed in FD lesions. Additionally, PDOs retained distinct constellations of genomic and metabolic alterations characteristic of FD. Histological evaluation further corroborated the fidelity of PDOs in recapitulating important phenotypic features of FD that underscore their pathophysiological relevance. Our findings represent meaningful progress in the field, as they open up the possibility for in vitro modeling of rare bone lesions in a three-dimensional context and may signify the first step towards creating a personalized platform for research and therapeutic studies.

## 1. Introduction

Fibrous dysplasia (FD) is a rare genetic disease that disrupts the physiological process of bone development to replace normal bone with fibro-osseous tissue. The disease manifests a wide spectrum of clinical signs and symptoms depending on the location and the biological behavior of the lesion. It may exhibit clinical aggression, even advancing into malignancy, or remain quiescent, going unrecognized [1]. With an estimated incidence of one in every thirty thousand individuals in the general population, FD poses a significant challenge due to its rarity [2]. Consequently, our understanding of the disease pathophysiology remains rather limited. The small number of patients and the inherent scarcity of data, coupled with insufficient funding, hampers progress in basic and preclinical research endeavors. Despite concerted efforts to address the shortage of biomarkers, the absence of clinically reliable and accurate indicators for FD continues to impede early detection and treatment. In this regard, establishing curated databases and developing preclinical models may be considered paramount for advancing our understanding of the disease processes, facilitating the subsequent development of therapeutic countermeasures against FD.

The evolution of bioengineering and stem cell research has ushered in an era of laboratory-grown miniature organs, termed “organoids,” since 2008 [3]. Initially focused on replicating various organs, organoid research has rapidly expanded to encompass a wide array of diseases [4,5,6,7,8]. Numerous strategies and methodologies have been developed and adopted to generate disease-specific organoid models, with patient-derived organoids (PDOs) emerging as a prominent exemplar. The breakthrough discovery of reprogramming adult human fibroblasts into induced pluripotent stem cells (iPSCs) has facilitated the generation of disease-specific iPSC lines from various patient cell types, including fibroblasts from individuals with Duchenne and Becker muscular dystrophy, Parkinson’s disease, Alzheimer’s disease, type I and II diabetes mellitus, Down syndrome, and cystic fibrosis [9,10,11,12]. However, the challenges and low success rate associated with iPSC derivation prompted the development of adult stem cell (aSC)-based organoid culture. Led by Hans Clevers and his colleagues, they discovered intrinsic stemness properties in aSCs when cultivated in extracellular matrix (ECM) hydrogel, supplemented with appropriate factors, enabling the successful generation of organoid cultures [13].

In contrast to iPSC-derived organoids, which incorporate components of the tri-lineage germ layers to closely mimic embryonic development, aSC-derived organoids exhibit limited differentiation potential, forming simpler structures derived from a single germ layer [14]. Nevertheless, unlike the delicate and sophisticated approach required for iPSC derivation and organoid establishment, aSC-derived organoids can be directly generated from tissue biopsies, allowing for the cultivation of disease-relevant organoids from patient tissues [15,16]. These aSC-derived organoids have proven to be valuable models for studying different types of fibrotic diseases. For instance, in 2013, Dekkers et al. demonstrated the successful establishment of primary cystic fibrosis organoids derived from rectal and duodenal biopsies [17]. Several studies ensued to expand this model system to include cystic fibrosis affecting different organs such as the lung [18], liver [19], stomach [20], and colon [21]. In recent developments, primary organoids derived from primary myelofibrosis and non-alcoholic steatohepatitis tissues have shown a remarkable ability to recapitulate the fibrotic microenvironment of their in vivo counterparts [22,23].

Despite the development of numerous organotypic models for fibrotic pathologic conditions, organoid-based modeling for fibrotic bone conditions like FD remains unexplored. Targeting fibrous bone tissues like FD lesions presents unique challenges due to difficulties in mirroring the distinctive structural properties of the FD extracellular matrix (ECM) and the complex biological characteristics, including interpatient heterogeneity and intralesional genetic and cellular diversity [24]. Retrospective analysis of FD lesions has revealed extensive inter- and intra-patient molecular and cellular heterogeneity [25]. Histopathological examinations have corroborated these findings, with case reports displaying sightings of various cell types, including fibroblastic stromal cells, osteogenic lineage cells, myeloid precursor cells, macrophages, osteoclasts, and T cells in biopsied FD tissues [26,27,28]. Given the plasticity and functional versatility of mesenchymal cells, osteoprogenitor cells in transitional phases, oscillating between fibrogenic and osteogenic cellular identities, are expected to constitute the lesional cell population.

To date, most relevant models of FD are based on transgenic or transplantation mouse models, which hold inherent limitations in the parameters possible for disease recapitulation [29,30,31,32]. PDOs offer a promising alternative by enabling the simulation of individual patient-specific genetic, molecular, and cellular profiles in an automated and scalable manner, facilitating their utility across a spectrum of research applications for basic research into drug discovery and testing.

In this study, we present an in vitro organotypic model capable of recapitulating distinct intrinsic and phenotypic properties of FD lesions. With the advent of scRNA-seq, we uncovered substantial transcriptional heterogeneity and diverse fibroblastic populations linked to pathological fibrosis. The organoids derived from primary cells of patient FD tissues exhibited a progressive increase in size and transparency that mirrored the pro-fibrotic microenvironment and cellular diversity observed in FD tissue counterparts. Additionally, PDOs demonstrated key molecular signatures and phenotypic features characteristic of FD. Our findings represent a foundational step toward developing in vitro 3D dysplastic bone lesion models, providing valuable insights into intrinsic and phenotypic aspects specific to the disease context for preclinical research.

## 2. Materials and Methods

### 2.1. Human Primary Specimens

Fresh tissue specimens were obtained from five patients diagnosed with craniofacial fibrous dysplasia who underwent surgery for lesion resections and normal craniofacial bone from healthy volunteers undergoing cosmetic facial bone contouring surgery. The collection and use of surgical specimens for research purposes was conducted under approved studies by the Institutional Review Board of Seoul National University Bundang Hospital (B-2111-718-302) and Samsung Medical Center (2021-12-025). The demographic and clinical characteristics of the donors are summarized in Table 1. Specimens were transported in PBS on ice and divided into three portions to be subjected to (1) snap freezing for DNA/RNA genomic extraction and tissue lysis for cAMP level measurement, (2) enzymatic digestion for cell extraction, and (3) 4% formaldehyde for histologic analysis.

### 2.2. Genomic DNA Extraction and Single Nucleotide Polymorphism (SNP) Genotyping

Cryopreserved specimens stored at −80 °C were immediately immersed in liquid nitrogen prior to processing for genomic DNA extraction. All specimens were reduced to fine powder by placing them in pre-made sterile aluminum pockets which were then struck with a hammer. Genomic DNA extraction was carried out using the DNeasy Blood & Tissue Kit according to the manufacturer’s instructions (Qiagen, Valencia, CA, USA). The extracted DNA (25–100 ng) was amplified in a 10ul reaction by standard polymerase chain reaction (PCR) with primers that generate a 351 bp product spanning the *GNAS* mutation (p.R201C/p.R201H) site. For each reaction, 10 μmol each of the primers 5′-GGACTCTGAGCCCTCTTTCC-3′ (forward) and 5′-CACAGCATCCTACCGTTGAA-3′ (reverse) were added, along with 2.5 units of Max DNA Polymerase (Doctor Protein, Seoul, S. Korea). The GNAS mutation status was determined by Sanger sequencing purified PCR products using the BigDye Terminator v3.1 Cycle Sequencing Kit (Applied Biosystems, Foster City, CA, USA) and analyzed with the Macrogen SNP analysis program v2.0 by the Macrogen Sequencing Facility (Macrogen, Seoul, S. Korea). The SNP genotyping was focused primarily on two targets, c.602G > A (p.R201H) and c.601C > T (p.R201C).

### 2.3. Specimen Dissociation and Cell Isolation

Fresh lesion specimens were finely minced into small particles until they reached the consistency of sand and digested with 2 mg/mL collagenase D solution (Roche, Mannheim, Germany) in basal medium Dulbecco’s Modified Eagle medium (DMEM; Gibco Life Technologies, Carlsbad, CA, USA) supplemented with 1% antibiotic-antimycotic solution, containing 10,000 U/mL penicillin and 10,000 U/mL streptomycin) (Life Technologies) for 4 h at 37 °C with gentle agitation. Digestion was terminated with fetal bovine serum (FBS; Life Technologies) and washed three times and resuspended in freshly prepared growth medium (DMEM supplemented with 10% FBS and 1% antibiotic-antimycotic solution). The resulting cell suspension was passed through a 70 μm cell strainer, pelleted by centrifugation at 300× *g*, resuspended with 5mL of 1× RBC Lysis Buffer (Invitrogen, San Diego, CA, USA), washed with DMEM, and pelleted for cryopreservation in cryopreservation medium (Nippon Zenyaku Kogyo, Fukushima, Japan) or cultured in a 75cm^2^ T-flask containing growth medium at a density of 3 × 10^5^ cells per flask in an atmosphere of 37 °C, 5% CO_2_, and 95% humidity. Any undigested residues were transferred to a 75 cm^2^ T-flask and maintained in growth medium for primary explant culture under normal culturing conditions. Approximately 2 weeks after explantation when outgrown cells reached near confluence, cells and tissue fragments were detached using 0.25% trypsin-EDTA (Gibco, Paisly, Scotland), washed, and resuspended in culture medium. Cells were collected from the suspension using a 70 μm cell strainer (PluriSelect, San Diego, CA, USA) and either cryopreserved or replated in fresh culture dishes.

Normal bone specimens consisted of marrow which was used to isolate bone marrow mesenchymal stromal cells (BMSCs). The marrow was scraped into the basal medium prepared as described above, followed by pipetting and serial passages through needles of decreasing diameter. Cells were treated with RBC Lysis Buffer prior to being either cryopreserved or plated into a 75 cm^2^ T-flask at a density of 3 × 10^5^ cells per flask containing growth medium (DMEM supplemented with 10% FBS and 1% antibiotic-antimyotic) and cultured until near confluence.

### 2.4. Single-Cell RNA Sequencing (scRNA-seq) and Data Pre-Processing

Cryopreserved cells dissociated from specimen digestion and cells obtained after two weeks of explant culture (2 × 10^6^ cells each) were delivered for scRNA-seq (Geninus Inc., Seoul, Republic of Korea). The Chromium system (10× Genomics, Pleasanton, CA, USA) was used to generate 50,000 read pairs per cell from the target population of 10,000 cells per sample, according to the manufacturer’s protocol. Raw sequencing reads in individual sample FASTQ files were aligned to the reference transcriptome GRCh38 (Ensembl-GENCODE 2020-A build) using the ‘cellranger count’ pipeline (CellRanger software version 7.1.0) (10× Genomics). Each sample’s individual Cell Ranger output filtered matrices were processed with the Seurat package (version 4.3.0.1) in the R software (version 4.0.3) (R Core Team, Vienna, Austria). After importation of the matrices in Seurat using the ‘Read10X()’ function, cells with fewer than 200 or more than 6000 expressed genes or with a percentage of mitochondrial DNA genes over 15% were filtered. Heterotypic doublets (and to a lesser extent homotypic doublets) occurring during cell encapsulation into droplets were removed using the R DoubletFinder package (version 2.0.3), as described in https://github.com/chris-mcginnis-ucsf/DoubletFinder (accessed on 19 April 2024). Finally, ambient RNA was removed using the R package SoupX (version 1.6.2) (Genome Research Ltd., Saffron Walden, UK) to remove cell-free mRNA contamination in the droplet using information from the Cell Ranger output raw matrices. Individual samples’ Seurat objects were merged and normalized. Briefly, gene counts for each cell were divided by the total counts for the cell and multiplied by a scale factor of 10,000, then natural log-transformed after adding 1 in order to avoid taking the log of 0. The top 2000 highly variable genes were then selected from this normalized expression matrix using the ‘vst’ method. Counts from the selected highly variable genes were then scaled, using the ‘vars.to.regress’ option in order to regress the signals from mitochondrial or ribosomal genes, followed by a linear dimensionality reduction through principal component analysis (PCA). Integration of the three FD samples was then performed to remove the batch effects using the R package Harmony (version 0.1.1) (https://github.com/immunogenomics/harmony2019, accessed on 19 April 2024) based on the top 20 PCA components identified in the previous step.

### 2.5. Cell-Clustering and Cluster Annotation

The integrated joint embedding from Harmony was used to cluster the cells using the Seurat package ‘FindClusters()’ function, using the Louvain algorithm to perform the graph-based clustering. The non-linear dimensional reduction ‘uniform manifold approximation and projection’ (UMAP) technique was used to visualize the identified clusters as a map. Differentially expressed genes (DEGs) with high cluster specificity were then selected using the ‘FindAllMarkers()’ function in Seurat using the ‘MAST’ test. The cluster top DEG genes as well as the expected cell-type canonical markers from the literature were used to annotate the clusters (described in Table S1). After quality control, doublets and ambient RNA removal, gene counts and percentage calculations of mitochondrial RNA-based filtering, a total of 30,030 cells were kept in Seurat for further analyses (FD3: 8288 cells, FD4: 9774 cells, and FD5: 11,968 cells). The average number of UMIs per cell was higher for the FD4 and the FD3 samples than for the FD5 sample (FD4 = 14,918; FD3 = 14,163, and FD5 = 8309), with the mean number of detected genes mirroring this effect (Figure S1).

### 2.6. Organoid Development and Culture

FD- and normal control-derived cells (5 × 10^4^ cells) at P0 were counted and resuspended in 10 μL of growth medium prior to being combined with Corning^®^ Matrigel^®^ Matrix for Organoid Culture (BD Biosciences, San Jose, CA, USA) at a 1:1 volume ratio on an ultra-low attachment 6-well plate (Stemcell Technologies, Vancouver, BC, Canada). The plate was incubated at 37 °C and 5% CO_2_ for 30 min to polymerize the drop. The drop was cultured in growth medium for 5 days before treating with 10 nM of parathyroid hormone (PTH) to stimulate cAMP production and osteogenic induction (OI) medium (DMEM supplemented with 10% FBS, 10 nM dexamethasone, 50 μg/mL ascorbic acid, 10 mM sodium β-glycerophosphate, and 1% antibiotic-antimycotic) [33]. A total of 25 PDOs were generated per patient. Among these, 10 PDOs were cultured in growth medium, while the remaining 15 were cultured in OI medium for subsequent experiments.

### 2.7. Organoid Imaging and Quantitative Analysis

Images of organoids were taken at day 21 and 28 using inverted microscopes, one in standard mode and the other in digital mode using the CMOS imaging sensor (KOPTIC HK6.3E3S), to observe for growth and morphological changes. Organoid size was determined by measuring the largest cross-sectional area, and organoid transparency was measured by calculating the mean and standard deviation of average white pixels, max intensity, and full-width half maximum (FWHM), using Image J software (Version 1.54i).

### 2.8. RNA Extraction and RT-qPCR

The total RNA was extracted from organoid and tissue specimens using RNA-Trizol Reagent (Thermo Scientific, Waltham, MA, USA), and cDNA was synthesized using the cDNA Synthesis Kit (Thermo Scientific). The mRNA expressions were quantified by real-time PCR using Power SYBR Green^®^ PCR Master Mix in a QuantStudio™ 7 Flex PCR Systems (Applied Biosystems, Waltham, MA, USA). Primers are listed in Table S2.

### 2.9. cAMP Assay

Cryopreserved tissue specimens were immediately submerged in liquid nitrogen and transferred to sterile aluminum pockets. They were then pulverized into fine powder using a hammer. Both tissue powders and organoids were resuspended in lysis buffer and subjected to sonication on ice to ensure complete lysis for the quantification of cyclic AMP (cAMP) levels using the cAMP Direct Immunoassay Kit (ab65355; Abcam, Boston, MA, USA), according to the manufacturer’s instruction. The assay was conducted in triplicate (three wells per sample), and the absorbance was measured at a wavelength of 450nm by the SpectraMax iD3 microplate reader (Molecular Devices, San Jose, CA, USA).

### 2.10. Histology

Tissue specimens underwent fixation in 4% paraformaldehyde overnight before being processed for paraffin embedding. Prior to processing, FD lesion and bone tissue specimens were decalcified in 10% EDTA until they reached the appropriate softness for full face sectioning, after which they were embedded in paraffin. Paraffin blocks were then sectioned into conventional 5 μm-thick slices, which were subsequently processed for hematoxylin and eosin (H&E) staining, Masson’s trichome staining, and von Kossa staining under standard protocols.

Organoids were fixed in 4% paraformaldehyde for 4 h before being embedded in HistoGel™ (Thermo Scientific) following the manufacturer’s instructions. Following a second fixation in 4% paraformaldehyde overnight at 4 °C, the HistoGel-encapsulated organoids were embedded in paraffin, sectioned, and routinely processed for H&E staining, Masson’s trichrome staining, and von Kossa staining. The organoids were sufficiently soft to undergo sectioning without requiring decalcification.

### 2.11. Immunofluorescence

Tissue sections underwent epitope retrieval using sodium citrate buffer, pH 6.0 (Enzynomics, Seoul, Republic of Korea), heated to boiling point in a microwave for 15 min, followed by cooling to room temperature (RT) for 30 min in a lukewarm bath. Subsequently, the sections were washed twice with PBS for 10 min each. Afterward, they were blocked in blocking reagent (SantaCruz Biotechnology, Santa Cruz, CA, USA) at RT for 30 min and then incubated overnight at 4 °C with appropriate primary antibodies: anti-collagen 1 (COL1), anti-alkaline phosphatase (ALP), and anti-sclerostin (SOST) for fluorescence-conjugated antibodies or anti-KI67, anti-alpha smooth muscle actin (α-SMA), and anti-transforming growth factor beta (TGF-β1) for non-fluorescence conjugated antibodies (all from Santa Cruz, diluted at 1:100 in blocking reagent). After washing with PBS three times for 10 min each, sections stained with fluorescence-conjugated antibodies were directly mounted using Vectashield mounting medium with DAPI (Vector Laboratories, Burlingame, CA, USA). Meanwhile, those stained with non-fluorescence conjugated antibodies were incubated with fluorescence-conjugated Alexa 488- or 594-conjugated goat anti-mouse or rabbit secondary antibodies (from Abcam, diluted at 1:250 in blocking reagent) for 1 h at RT before repeating the washing and mounting steps in Vectashield mounting medium with DAPI. Final histological images were taken using a Zeiss LSM800 confocal microscope after curing the mounting medium overnight.

Whole-mount stainings were performed for organoids to visualize the architecture and immunolocalization of proteins in three dimensions. Initially, whole organoids were fixed in 4% paraformaldehyde overnight at 4 °C, permeabilized with 0.5% Triton-X100 for 1 h, and subsequently blocked with blocking reagent for 2 h. Following the same protocol as for tissue sections, organoids were then incubated overnight at 4 °C with gentle agitation using either fluorescence-conjugated primary antibodies, such as anti-COL1, anti-ALP, and anti-SOST (all from Santa Cruz, diluted at 1:100 dilution in blocking reagent), or non-fluorescence conjugated primary antibodies, such as anti-KI67, anti-alpha smooth muscle actin (α-SMA), and anti-transforming growth factor beta (TGF-β1) (all from Santa Cruz, at 1:100 dilutions in blocking reagent). After extensive washing with PBS, six times for 10 min each at RT, organoids stained with fluorescence-conjugated antibodies were directly resuspended in Vectashield mounting medium with DAPI (Vector Laboratories, Burlingame, CA, USA) and dispensed on a confocal dish with a coverslip on top. Organoids stained with non-fluorescence conjugated antibodies were incubated with fluorescence-conjugated Alexa 488- or 594-conjugated goat anti-mouse or rabbit secondary antibodies (from Abcam, diluted at 1:250 in blocking reagent) for 1 h at RT before repeating the washing process and mounting in Vectashield mounting medium with DAPI. The mounting medium was cured overnight before imaging using a Zeiss LSM800 confocal microscope.

### 2.12. Statistical Analysis

Statistical analysis and data representation were performed using GraphPad Prism 8 (GraphPad Software, San Diego, CA, USA, Version 8.0.2). The statistical differences were determined by one-way ANOVA or Kruskal–Wallis test for multiple pairwise comparisons, where appropriate. All quantitative results are presented as the mean ± standard deviation of five independent experiments, each performed in triplets, if not noted otherwise. A *p*-value < 0.05 was considered statistically significant.

## 3. Results

### 3.1. Integrating Patient’s Biology into Organoid Model System

Taking into account the limited patient pool (*n* = 5) and their relatively young ages (18, 19, 25, 18, 18), this study aimed to establish a patient-derived organoid model from surgically resected FD-lesion specimens. All patients presented with active lesions in the craniofacial region requiring surgical intervention. While previous studies often resorted to bone marrow stem cells (BMSCs) obtained from FD patients’ bone marrow aspirates or bioengineered BMSCs for disease modeling [30,34,35,36], procuring marrow from young patients, such as those in our study, for research purposes, poses risks of bleeding, infection, and pain, rendering this option impractical and unethical. Considering that the fibrotic tissue of FD lesions arises from a bone marrow abnormality, direct use of the lesional tissue seemed feasible. Supported by past research demonstrating successful reproduction of FD-like characteristics in mouse models using lesion-derived cells, this study proposes a protocol for organoid generation from accessible tissue sources, namely resected FD-lesion specimens, which requires no additional invasive measures for harvest [32,37,38,39].

**Figure 1:**
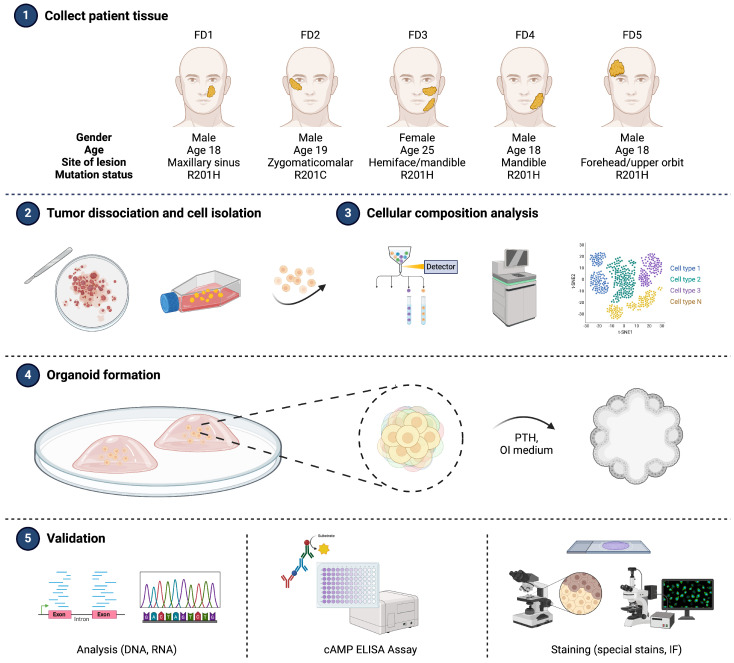
Schematic overview of the patient-derived organoid (PDO) development workflow. (Step 1) Specimens are collected from five patients with fibrous dysplasia who underwent surgical resection. (Steps 2 and 3) Specimens are dissociated as individual cells for characterization of specific cell types. (Step 4) FD tissue-derived cells are embedded in Matrigel and differentiated into organoids. (Step 5) These organoids are validated using methods including sequencing, ELISA, and staining. FD, fibrous dysplasia; PTH, parathyroid hormone; OI, osteogenic induction; cAMP, cyclic adenosine monophosphate; IF, immunofluorescence.

The study design proposed is outlined in Figure 1. Tissue specimens from five patients diagnosed with FD were promptly collected in the operating room and placed on ice (step 1). Tissue resections were performed using stereotactic, image-guided saw and osteotome followed by tangential shaving. Any different tissue components aside from the FD lesion were removed prior to subjecting it to mincing and enzymatic digestion for cell isolation (step 2). Individual cells isolated from the specimen were analyzed using scRNA-seq to characterize the cellular composition of tissue samples (step 3) and the remaining cells were propagated for characterization and organoid development (step 4). To determine the relevance of the organoids to FD, derived organoids and corresponding patient tissues were subjected to SNP sequencing for GNAS mutation, RT-qPCR, and ELISA for molecular signatures, as well as histological evaluation for recapitulated pathological features (step 5).

### 3.2. Single-Cell Analysis Unveils Pro-Fibrotic Transcriptional Signatures and Fibroblastic Cellular Heterogeneity in FD Lesions

**Figure 2:**
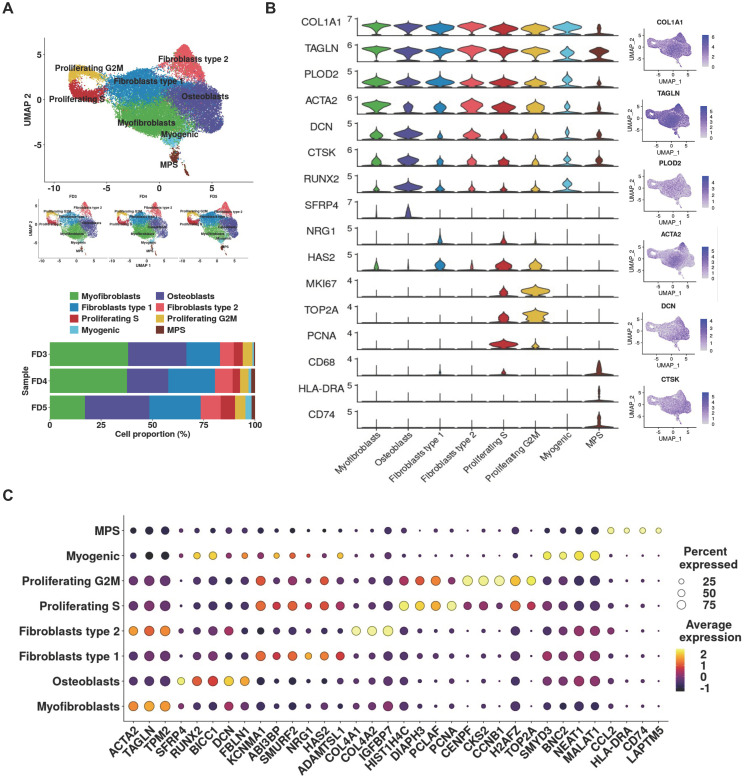
Single-cell transcriptome profiling of cell isolates from FD lesion. (**A**) Combined and individual UMAP projections for the eight cell-type clusters and relative proportion of each cluster across the 3 FD-lesion specimens analyzed. (**B**) Violin plots representing the normalized expression of signature genes identified as common or uniquely expressed for different cell-type clusters. Expression of selected common genes projected onto UMAP visualization. (**C**) Dot plots representing the top DEGs of each cell-type cluster. The dot size encodes the proportion of gene-expressing cells within the cluster, and the color represents normalized average expression.

To unravel the molecular and cellular heterogeneity of FD lesions, we initiated scRNA-seq analysis on three lesion specimens obtained from FD patients (FD3, FD4, and FD5). Using UMAP projection to visualize single-cell gene expression profiles, we identified eight major clusters (Figure 2A). Notably, a continuum of canonical pro-fibrotic markers collagen type 1 alpha 1 (COL1A1), transgelin (TAGLN), procollagen-lysine,2-oxoglutarate 5-dioxygenase 2 (PLOD2), and smooth muscle alpha-actin (ACTA2) was detected in all clusters, albeit with variable expression levels (Figure 2B). To refine our annotations and address overlapping or ambiguous genes, joint analyses of cell-type canonical markers from Table S1 and overexpressed DEG profiles (Figure 2C) were implemented to isolate unique identifier genes for each cluster. The following cell types were identified: myofibroblasts (green), osteoblasts (purple), fibroblasts type 1 (blue), fibroblasts type 2 (pink), proliferating S cells (red), proliferating G2M cells (yellow), myogenic cells (light blue), and lastly, mononuclear phagocyte system (MPS) cells (brown).

Minor interpatient variability was observed in cell-type proportions despite the uniform cellular composition (corresponding cell counts and proportions are available in Table S3). Specifically, the FD3 and FD4 samples exhibited a higher proportion of myofibroblasts (38.1 and 37.6%, respectively) compared to 17.2% in the FD5 sample. Conversely, osteoblasts accounted for the highest proportion of cells in the FD5 sample (31.3%). Overall, the proportions of myogenic and MPS cells were relatively smaller, particularly in the FD3 sample, where only 51 and 66 cells (0.6 and 0.8% of the total cells) were assigned to the myogenic and MPS clusters, respectively. These differences may be attributed to factors such as the lesion site and its status of lesion development and aggression. Unfortunately, information regarding the time of lesion initiation and the pathological and biological behavior of the lesion in FD patients was not available. However, upon closer examination of the lesion sites, it was observed that lesions in FD3 and FD4 samples were located in the lower jaw, whereas the lesion in FD5 was situated in the forehead and upper orbit. This discrepancy in lesion site potentially explains the differences in the proportions of myofibroblasts and osteoblasts observed among the samples.

The top differentially expressed genes corresponding to each identified cell type revealed exclusive sets of genes characteristic of the proposed cell lineage. The top 10 most differentially expressed genes are provided in File S1. Myofibroblasts expressed high levels of genes encoding *ACTA2*, a marker for smooth muscle actin; *TAGLN*, a small acting-binding protein involved in smooth muscle differentiation; and tropomysin 2 (*TPM2*), a contractile protein regulating muscle contraction [40]. Osteoblasts demonstrated exclusive enrichment for secreted frizzled related protein 4 (*SFRP4*), a critical protein primarily expressed by the osteoblast lineage to modulate bone formation and remodeling processes, as well as other early osteoblast markers, including runt-related transcription factor 2 (*RUNX2*), BicC family RNA binding protein 1 (*BICC1*), decorin (*DCN*), and fibrillin 1 (*FBLN1*) [41,42].

Fibroblasts type 1 expressed potassium calcium-activated channel subfamily M alpha 1 (*KCNMA1*) [43], ABI family member 3 binding protein (*ABI3BP*) [44], and SMAD specific E3 ubiquitin protein ligase 2 (*SMURF2*) [45], mesenchymal markers of cellular senescence, and a disintegrin and metalloproteinase with thrombospondin motif type 1 (*ADAMTSL1*) [46], a discerning marker specific for fibroblasts and stromal cells of fibroblast lineage. This cluster also displayed increased expression levels of relevant phenotypic markers of fibroproliferative disorders, such as neuregulin 1 (*NRG1*), encoding a membrane glycoprotein identified as being expressed by hypertrophic scar-derived fibroblasts [47], and hyaluronan synthase 2 (*HAS2*), an enzyme critical in promoting fibrosis via cellular hyaluronan production in various fibrotic diseases [48]. Based on these findings, we defined the cluster as a population of stromal cells/fibroblasts relevant to FD pathology. A second cluster of fibroblasts, fibroblasts type 2, expressed common genes associated with contractility (*ACTA2*, *TAGLN*, and *TPM2*), as well as the top three differentially expressed genes associated with the fibrotic matrisome, (collagen type IV alpha 1 (*COL4A1*), collagen type IV alpha 2 (*COL4A2*), and insulin-like growth factor binding protein 7 (*IGFBP7*)) [49]. It is known for fibroblasts to adopt myofibroblast phenotypes upon activation by stress or injury [50]. A similar population of α-SMA positive cells expressing collagenous matrisome genes *COL4A1* and *COL4A2* were discovered in idiopathic pulmonary fibrosis (IPF) and lung cancer tissues [49]. These shared similarities led to our interpretation of the cluster as a population of pathological myofibroblasts.

Two distinct clusters of proliferating cells expressed common bone metabolic markers *KCNMA1*, *ABI3BP*, and the *SMURF2* characteristic of stromal cells. However, they also exhibited high expression of S and G2M specific cell-cycle-related genes, proliferating cell nuclear antigen (*PCNA*) for S, and DNA topoisomerase II alpha (*TOP2A*), centromere protein F (*CENPF*), and marker of proliferation KI67 (*MKI67*) for G2M [51] as its unique distinguishing feature. The robust proliferative potentials observed in these clusters are widely recognized as fundamental behavioral traits of pathological fibrosis, serving as pivotal drivers for excessive ECM deposition, thereby indicating the pro-fibrotic nature of these cells.

Moreover, myogenic cells expressed genes associated with myogenic differentiation, including SET and MYND domain-containing 3 (*SMYD3*) [52], basonuclin zinc finger protein 2 (*BNC2*) [53], nuclear paraspeckle assembly transcript 1 (*NEAT1*) [54], and metastasis-associated lung adenocarcinoma transcript 1 (*MALAT1*) [55]. Additionally, a small cluster of MPS cells uniquely expressed the major histocompatibility complex, class II, DR alpha (*HLA-DRA*) and cluster of differentiation 74 (*CD74*) [56], markers for dendritic cells, along with *CD68* [57], a canonical marker for macrophages.

### 3.3. Organoid Generation and Morphological Assessment

**Figure 3:**
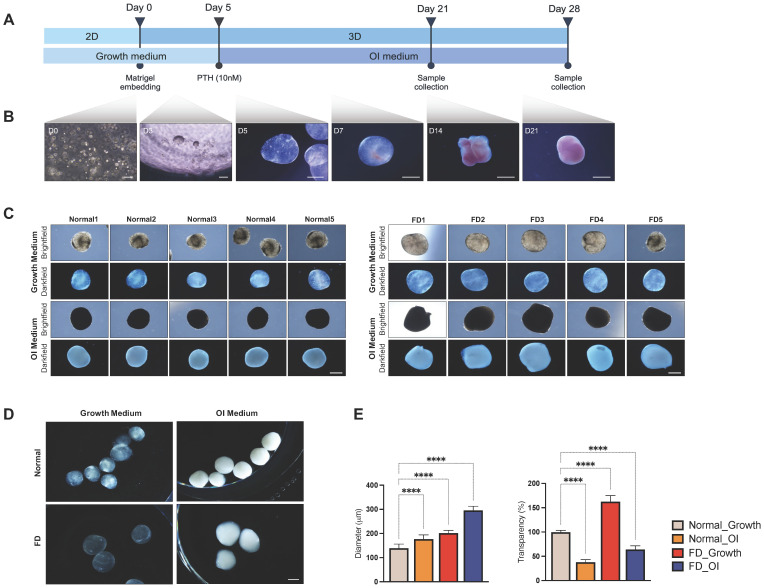
Production of PDOs. (**A**) Experimental scheme of protocol used to generate PDOs. (**B**) Morphological changes tracked on day 0, 3, 5, 7, 14, and 21 during the culture with Matrigel. (**C**) Brightfield and darkfield illumination images of organoids grown under growth culture and OI conditions on day 21 from five independent experiments. (**D**) Microscopic imaging using digital sensor of organoids on day 28. (**E**) Quantification of the diameter and the transparency of organoids. The asterisks denote significant differences (**** *p* value < 0.0001, one-way ANOVA). Scale bar, 100 μm. PTH, parathyroid hormone; OI, osteogenic induction; FD, fibrous dysplasia.

Organoids were generated using a three-dimensional culture protocol (Figure 3A). Patient-derived cells were embedded in droplets of Matrigel and cultured for 5 days in growth medium before being transferred to OI medium, consisting of ascorbic acid, β-glycerophosphate, and dexamethasone, and the cAMP stimulant, PTH peptide. Over a period of 2 weeks, cells underwent self-assembly, transitioning from irregular shapes to more rounded morphologies (Figure 3B). Organoids were maintained in OI medium for a minimum of 28 days with medium changes every 3 days. However, exceptions were made for cell constructs cultured in growth medium.

Observation of organoids cultured in either growth or OI medium, under brightfield and darkfield illumination, displayed distinct morphological differences between patient- and normal control-derived organoids with respect to their size and transparency (Figure 3C). These disparities were consistently observed between PDOs and normal control-derived organoids under both growth and OI conditions by day 21. By day 28, the differences in size and transparency became more pronounced (Figure 3D,E). Darkfield microscopy showed that PDOs cultured under growth conditions were highly transparent, while those cultured under OI conditions developed moderate opacity with less-distinct outer edges. In contrast, organoids from normal controls displayed a more compact architecture with irregular cellular aggregation under growth conditions. Under OI conditions, the distribution of density became more homogenized, resulting in full opacity. Organoids grown under OI conditions bore greater resemblance to the corresponding FD or normal-control-tissue counterparts, and were selected for further characterization and evaluation.

### 3.4. PDOs Exhibit Enrichment of FD-Relevant Gene Signatures and Cellular Microenvironment

**Figure 4:**
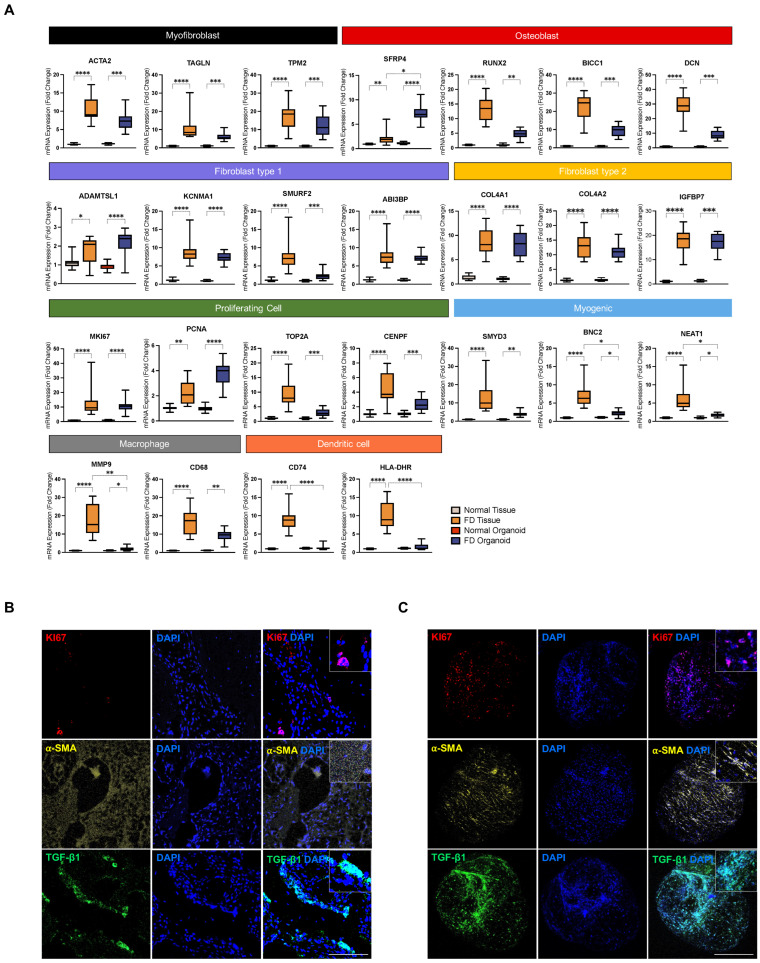
Analysis of cellular, genetic, and protein signatures of parent tissues and derived organoids. (**A**) Boxplots of relative mRNA expressions of selected signatures from each cell-type cluster, extracted from the scRNA-seq dataset, in parent tissues and derived organoids. Boxes indicate the interquartile range, separated by median line. Whiskers indicate the maximum and minimum values. The asterisks denote significant differences (**** *p* value < 0.0001, *** *p* value < 0.001, ** *p* value <0.01, * *p* value < 0.05, Kruskal–Wallis test). (**B**) Immunofluorescent images of FD tissue and (**C**) patient-derived organoids for proliferating cell marker (KI67; red), activated fibroblast marker (α-SMA; yellow), and pro-fibrotic marker (TGF-β1; green). Scale bar, 100 μm.

The gene signatures for interrogating the cellular components of PDOs were derived from our scRNA-seq dataset. Enrichment for the three major cell constituents—myofibroblasts, osteoblasts, and stromal fibroblasts—was determined using specific markers: TAGLN, TPM2, and ACTA2 for myofibroblasts; SFRP4, RUNX2, BICC1, and DCN for osteoblasts; and ADAMTSL1, KCNMA1, SMURF2, and ABI3BP for stromal fibroblasts (Figure 4A). Normal control-derived organoids served as the comparison group. RT-qPCR confirmed a significant upregulation of mRNAs encoding markers for myofibroblast, osteoblast, and stromal fibroblast, suggesting conserved populations of all three cell types. Intriguingly, a notable increase in SFRP4 expression was observed in PDOs rather than in FD tissues. SFRP4 is known to play a pivotal role in osteoblast differentiation, but its overexpression has been linked to the inhibition of bone formation by suppressing osteoblast proliferation and maturation [58]. Therefore, its prominent expression in PDOs may be attributed to the differentiated state of the PDOs, as SFRP4 is typically highly expressed in mature osteoblasts [59].

Additionally, the presence of proliferating population of cells within the organoids was confirmed by assessing cell-cycle and proliferation markers MKI67, PCNA, TOP2, and CENPF, in comparison to their FD-tissue counterparts. The findings yielded coherently high expression levels in both PDOs and corresponding patient tissues, confirming their enrichment for proliferating cells. Importantly, PDOs exhibited significant enrichment in PCNA expression. Elevated PCNA expression is strongly correlated with higher cell-proliferation rates, owing to increased activity of cells in the G1/S phase [60,61]. This observation suggests that PDOs display robust proliferative cellular behavior, which can potentially elicit a physiologically relevant pro-fibrotic phenotype. In line with these results, immunofluorescence staining of the FD tissue and PDO for proliferation marker, KI67, fibroblast activation marker, α-SMA, and pro-fibrotic marker, transforming growth factor beta-1 (TGF-*β*1), further supported the presence of proliferating cells positive for KI67, activated fibroblasts positive for α-SMA, and a TGF-*β*1-rich pro-fibrotic microenvironment within FD lesions, all of which were recapitulated in PDOs (Figure 4B,C). It can be inferred from these findings that potent niche factors orient cells towards a fibroproliferative state, which in turn contributes to the abnormal deposition of ECM and collagen within FD lesions. Further analysis of myogenic and macrophagic markers indicated the retention of both cell types within PDOs. Additionally, dendritic cells, the smallest cluster observed in the FD lesion, may be present in small amounts in PDOs, based on its minimal marker expression. Collectively, our findings demonstrate that PDOs preserve the cellular composition and harbor disease-relevant cellular microenvironment in a similar way to that of the tissue of origin.

### 3.5. PDOs Recapitulate the Genetic Abnormality, Metabolic Alteration, and Functional Defects of Corresponding FD Tissues

**Figure 5:**
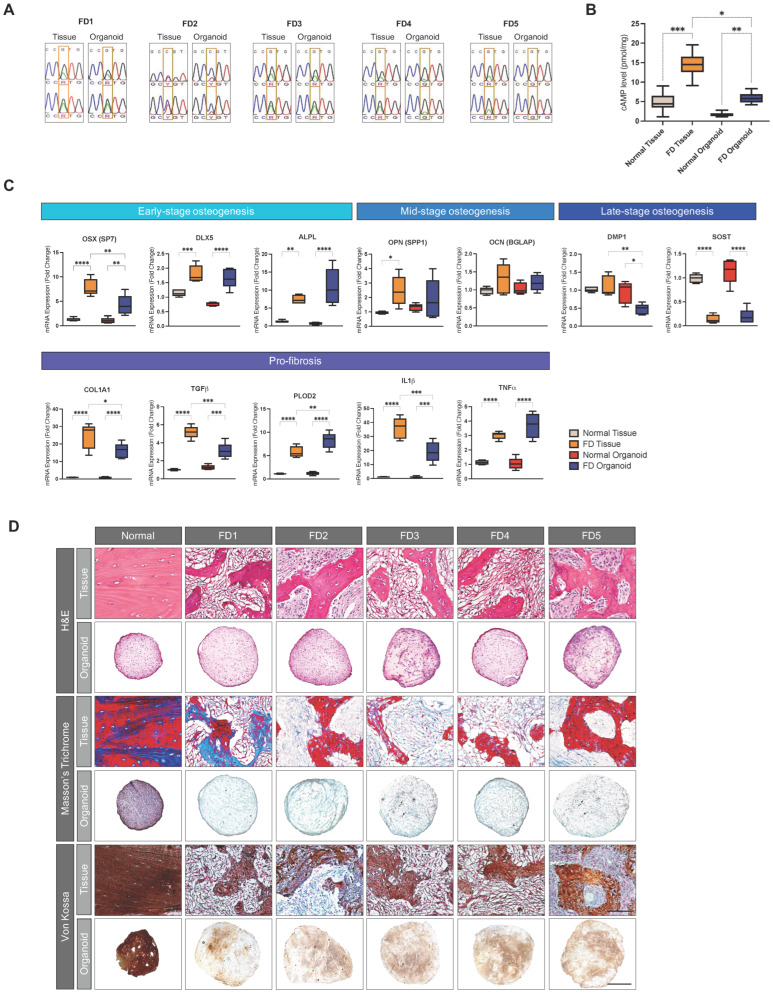
Histological evaluation of parent tissues and derived organoids. (**A**) Genomic sanger sequencing for GNAS mutation in parent tissues and derived organoids. (**B**) Boxplot of cAMP cellular content in parent tissues and in total cell lysate of derived organoids. (**C**) Boxplots of mRNA expressions of selected osteogenic and pro-fibrotic markers in parent tissues and derived organoids. Boxes indicate the interquartile range, separated by median line. Whiskers indicate the maximum and minimum values. The asterisks denote significant differences (**** *p* value < 0.0001, *** *p* value < 0.001, ** *p* value <0.01, * *p* value < 0.05, Kruskal–Wallis test). (**D**) H&E staining, Masson’s trichome staining, and von Kossa staining. Masson’s trichrome staining: blue, collagen content. Von Kossa staining: brown, calcium mineral deposits. Scale bar, 100 μm.

Despite the ambiguity surrounding the exact underlying etiology of FD, the prevailing consensus reached implicates the importance of GNAS mutation in driving the constitutive overproduction of cyclic adenosine monophosphate (cAMP) in dysplastic cells, leading to increased fibrous ECM deposition and reduced mineralization [62,63]. We evaluated the GNAS mutation status in PDOs using SNP sequencing (Figure 5A). Four out the five PDOs expressed patient tissue-specific GNAS mutations, indicating potential conservation of the mutation. To assess the translation of the mutation into the cellular phenotype distinct to FD in PDOs, such as cAMP overproduction, we measured the intracellular cAMP concentration. PDOs showed significantly elevated levels of cAMP compared to the normal controls, consistent with the parent -issue counterparts (Figure 5B).

Further analysis of the functional aspects of PDOs using RT-qPCR and special stains revealed the recapitulation of central disease features involving excessive collagenous matrix deposition in place of mineral contents (Figure 5C). mRNA assessment of bone markers for different stages of osteogenesis and pro-fibrotic markers presented increased early-stage osteogenic markers osterix (OSX), distal-less homeobox 5 (DLX5), and alkaline phosphatase (ALPL), and significantly decreased late-stage markers dentin matrix acidic phosphoprotein 1 (DMP1) and sclerostin (SOST), along with a marked rise in pro-fibrotic markers COL1A1, TGF-*β1*, PLOD2, interluekin 1 beta (IL-1*β*), and tumor necrosis factor alpha (TNF-α), which were highly correlative to the expression patterns observed in parent FD tissues.

Histological findings were in accordance with the mRNA expressions (Figure 5D). H&E staining showed preservation of the cellularity of parent tissues in PDOs. Despite non-discrete architectural features, Masson’s trichrome staining demonstrated a homogenous blue stain in PDOs, signifying collagen presence consistent with the collagenous ECM distributed within the fibrous stroma of the parent FD tissue. Additionally, von Kossa staining exhibited an evenly distributed strong reactivity to large calcium deposits in normal control-derived organoids, contrasting with the weaker, patchy von Kossa reactivity in PDOs, reflecting reduced calcium mineral content in FD. Together, these findings demonstrate the capacity for PDOs to recapitulate genetic and phenotypic characteristics specific to FD.

### 3.6. PDOs Display Non-Aligned, Isotropic Distribution Patterns of Extracellular and Cellular Components

**Figure 6:**
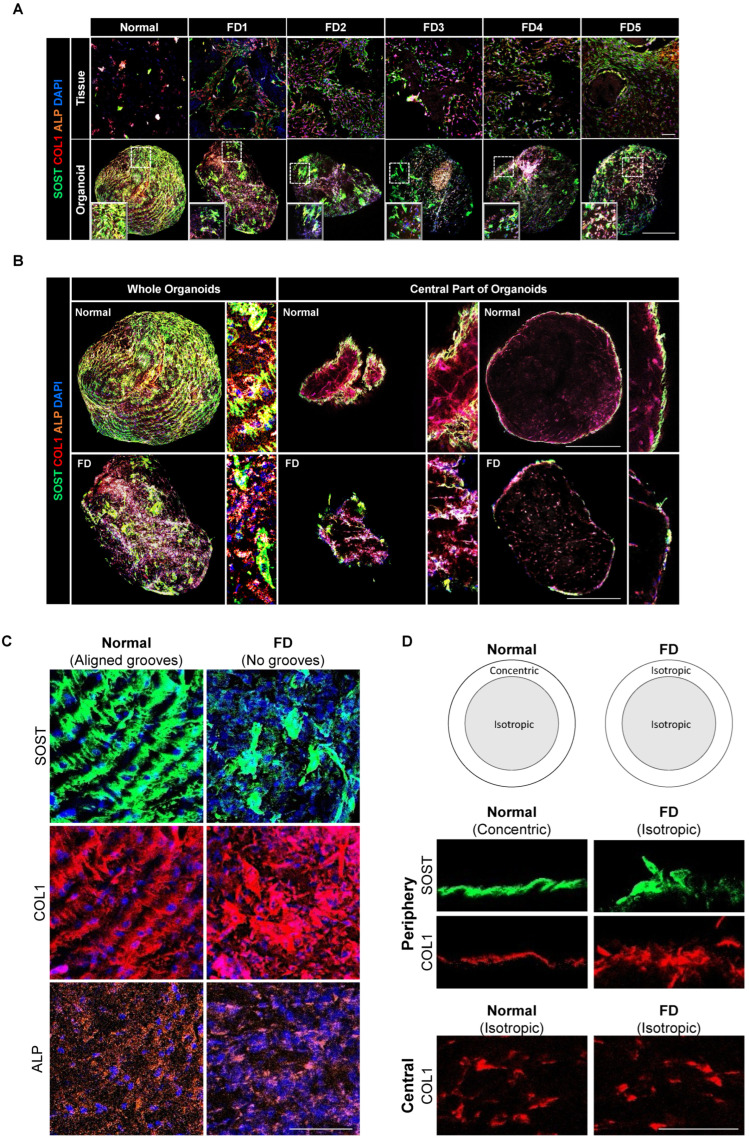
Functional characterization of PDOs. (**A**) Multiplexed immunohistochemistry images of parent tissues and derived organoids with SOST (green), COL1 (red), and ALP (orange). (**B**) Representative three-dimensional images of the whole organoid and central part of the organoids. Scale bar, 100 μm. (**C**) Images depicting the distribution of marker expressions along the organoid periphery and (**D**) the concentric or isotropic arrangements in the peripheral and central regions of the organoids. Scale bar, 20 μm.

To assess the expression and distribution of key FD markers, whole-mount organoids and sectioned parent tissues were multiplexed for collagenous matrix marker COL1, mature osteocyte marker SOST, and early osteoblast marker ALP (Figure 6A). Consistent with previous observations, prominent expressions of COL1 and ALP were observed in the fibrous stroma of FD tissues, while SOST was localized to the margins of the bony trabeculae [30,64]. Similarly, PDOs showed positive staining for COL1 and ALP, with a higher intensity of COL1 expression and limited, sporadic staining of SOST.

Z-stacking across multiple focal planes revealed distinct spatial arrangements and distribution patterns of these marker expressions in the peripheral and central regions of the organoids (Figure 6B). In both PDOs and normal control-derived organoids, SOST was confined to the periphery, while COL1 and ALP expression spanned the entire surface and interior of the organoids. The unique distribution patterns of SOST and COL1 served as discerning features between PDOs and normal control-derived organoids. In normal control-derived organoids, COL1, in tandem with SOST, formed parallel radial grooves across the entire periphery, creating multi-layered, organized structures. In contrast, PDOs displayed irregular, randomly oriented distributions (Figure 6C). ALP expression within the collagenous matrix was notably less pronounced in PDOs compared to normal control-derived organoids.

Examination of a single layer of the organoids revealed varying physical microfeatures in orientation. In normal control-derived organoids, COL1 exhibited anisotropy in the concentric direction to form a well-defined layer, while PDOs presented a textured planar surface of isotropic COL1 deposition (Figure 6D). SOST aligned with COL1 to exhibit either concentric or isotropic patterns. The central regions of both PDOs and normal control-derived organoids showed an isotropic annulus of COL1 encircling the entire interior. These findings suggest differing spatial arrangements and distribution patterns of extracellular and cellular components in PDOs compared to normal control-derived organoids, suggesting variations in structural organization.

## 4. Discussion

The FD bone lesion, arising from a single genetic defect of GNAS, manifests to present extraordinarily complex and unique physiological and pathological properties distinct from many other bone-related diseases. This complexity makes FD one of the more challenging, yet promising, disease targets to model. With new paradigms for understanding disease biology in par with evolving technologies that provide the edge necessary for effective disease modeling and therapeutic development, rare diseases no longer seem insurmountable. We herein intended (i) to widen the appreciation for the complexity behind FD biology by providing the first landscape of the molecular and cellular heterogeneity of FD lesions and (ii) to establish a PDO model capable of recapitulating cellular, genetic, and molecular signatures, as well as the functional features of FD bone lesions.

Given the nature and goals of this study, possible hurdles that were expected to be encountered during our pursuit were the following: FD is a fibro-osseous lesion with a heterogeneous cellular and genetic makeup; it is a genetic disease characterized by various GNAS-mutant isoforms observed across patients; and it shows a dynamic tissue comprised of a wide spectrum of normal and mutant cells in variable proportions. Despite attempts to portray the heterogeneity and intricacies of FD in mouse models, a limited degree of biological similarity with humans and challenges in precisely controlling microenvironments remain as critical matters. These various aspects draw our attention to the importance of developing a robust model that can accurately portray the pathological and molecular features unique to FD. PDO offers an effective solution that can bridge the gap between existing mouse models and human cellular models. It allows for the creation of personalized biological systems amenable to a wide range of applications, including basic research, to expand our understanding of molecular mechanisms and disease pathology or drug discovery to develop patient-tailored precision and/or regenerative medicine.

While FD bone lesions have traditionally been thought to house dysplastic bone marrow mesenchymal/stromal cells that promote the pathological ECM deposition, the specific cell types involved and their contribution to FD pathogenesis remain poorly understood. Our scRNA-seq data provided valuable insights into the extensive transcriptional heterogeneity and the different fibroblastic cell types of FD lesions to offer new perspectives on disease pathology. Here, we identified eight cell types, among which myofibroblasts, osteoblasts fibroblasts, and proliferating cells comprised the major cell constituents. Some were previously un-annotated cell types with unique transcriptomic profiles for FD. Intriguingly, all cell types expressed overlapping transcriptional signature markers associated with fibroblastic and osteoblastic phenotypes (*COL1A1*, *RUNX2*, *TAGLN*, *ACTA2*, and *DCN*) (Figure 2B), to potentially implicate a common mesenchyme origin under the transition towards the fibroblastic phenotype.

Fibroblasts, known for their remarkable diversity and plasticity, are believed to play a significant role in the progression of fibrotic diseases [50,65,66]. Under appropriate conditions, fibroblasts in their resting state can adopt osteoblastic phenotypes, obscuring the lines of demarcation between fibroblasts and osteoblasts [67,68]. Various subtypes of resting fibroblasts have been identified, namely, myofibroblast, fibrogenic cells, smooth muscle cells, and mesenchymal stem/stromal cells. Upon exposure to an inflammatory stimulus, fibroblasts can activate and exhibit altered transcriptional profiles [69]. Even activated fibroblasts have demonstrated plasticity to interconvert into MSCs or their progeny, suggestive of the possibility for MSCs or osteoblasts to serve as precursors for activated fibroblasts [70]. The ubiquitous expression of fibroblast marker *COL1A1* and *PLOD2* across all clusters, along with the recurrence of mesenchymal markers *KCNMA1*, *ABI3BP*, and *SMURF2* and contractile markers *ACTA2, TAGLN*, and *TPM2,* advocates the possibility of a dynamic flux of fibroblast and stromal cells at various stages of transition from a quiescent to a reactive state within FD lesions (Figure 2B,C and Figure S2). The three predominant populations, identified as myofibroblasts, osteoblasts, and fibroblasts, according to the notion above, may hold the potential for interconversion.

Besides the three cell types identified, the authors posit that the proliferating S and G2M clusters present unique populations of proliferating stromal cells specific to FD. Genes encoding nuclear proteins such as *HIST1H4C*, *H2AFZ*, *CENPF*, and *CKS2*, which interact with cell-cycle regulatory proteins, were among the most highly expressed genes in these two clusters (Figure 2C, File S1). Of interest to us is the fact that these genes have been recognized as exclusive biomarkers for cell proliferation associated with a higher S and G2M score, suggesting functions in growth and cell division [71,72,73]. Moreover, *TOP2A* and *CENPF*, markers for cell-cycle regulation that were among the exclusively expressed genes in the proliferating S and G2M clusters, are reported to synergistically promote cell division, epithelial–mesenchymal transition (EMT), proliferation, and migratory phenotypes [74]. Additionally, other markers exclusive to the proliferating cells, such as *MKI67* and *PCNA*, have been implicated in fibrotic conditions, including IPF, hepatic fibrosis, and cancer [75,76,77]. The distinctive expression profiles of fibrosis-associated markers led to our putative annotation of proliferating cells being relevant to FD pathology.

The characterization of PDO cellular composition unveiled transcriptional signatures indicative of the retained presence of major cell types, i.e., myofibroblasts, osteoblasts, and fibroblasts. Additionally, highly proliferative stromal cells, as evidenced by enhanced expression of G1/S phase markers, were identified. Together, their collective expansion within an organotypic system may foster a pro-fibrotic microenvironment capable of eliciting fibrotic responses and vice versa through cell–cell and paracrine interactions. Positive staining for activated fibroblasts positive for α-SMA and proliferating cells positive for KI67, and a pro-fibrotic microenvironment rich in TGF-β1 in FD tissues, also emulated in PDOs, supports this possibility. While further molecular characterization is needed to elucidate cellular cross-talks and interactions using co-culture systems, increased expressions of pro-fibrotic markers *COL1A1*, *PLOD2*, *IL-1β*, and *TNF-α* at the mRNA level indicate a PDO microenvironment highly conducive to fibrosis.

Further analysis revealed specific features of FD recapitulated in PDOs. For instance, the deposition of collagenous rather than mineralized contents in PDOs with patient-specific GNAS mutation and elevated cAMP levels, hallmarking fibrotic events in FD, attests to the validity of this modeling approach. It is interesting to note that PDOs lacking the GNAS mutation sustained the fibrotic phenotype. Mutant cells, which constitute only a mere 20 percent of the total FD cell population [78], diminish over time due to restricted self-renewal and apoptotic tendencies, leading to quiescence [32,79]. Consequently, the limited population of mutation-bearing cells in PDOs is likely consumed by apoptosis and outnumbered by non-mutated cells for its eventual elimination. From this, we can deduce that even after the removal of mutated cells, the initially orchestrated pro-fibrotic environment in PDOs persists in inducing fibroblast phenotypes, hindering normalization towards the control phenotype.

Immunostaining for key extracellular proteins COL1 and SOST revealed distinct distribution patterns reminiscent of the bone microarchitecture specific to their respective sources. Sclerostin, a product of SOST released by mature osteocytes, is critical for normal bone formation [80]. The structured arrangement of SOST deposition in alignment with COL1 supports potential emulation of the spatial organization of osteocytes in coordination with collagen ECM orientation. Osteocytes are known to respond to the structural cues provided by the concentric collagen patterns of the ECM, resulting in preferential alignment and directed cellular migration [81,82]. The concentric, circumferential grooves of COL1 and SOST at the periphery of normal-derived organoids reflect a structured arrangement of osteocytes and collagen ECM, which is characteristic of the highly organized mineralized collagen fibrils and osteocyte alignments found in lamellar bone [83]. In contrast, the isotropic and random distribution of these proteins in PDOs suggests a lack of spatial organization typical of woven bone, a less-mature and more disordered form of bone architecture [84,85]. This pattern resembles the aberrant structural organization characteristic of FD, with disorganized and randomly oriented collagen fibrils and inconsistently aligned osteocytes, signaling a deviation from normal bone microarchitecture. The unique spatial registrations of extracellular and cellular components within the organoids lend further credibility to their biological functionality.

While these findings highlight the potential of PDOs to model certain FD-relevant features, this approach poses several limitations as well. First, being an in vitro organoid model based on primary cells from patient FD tissues, the organoids were unable to replicate the complex morphological and histological features of FD. Despite providing initial insights into 3D expansion of patient-derived cells in culture, addressing the recognized limitations of primary organotypic models necessitates the next step of in vivo transplantation of organoid explants to verify the formation of FD-like tissues. Second, a thorough transcriptional characterization of the profiles of individual cells comprising the PDOs is essential for cross-comparison with datasets of the parent tissues to delineate the transcriptomic profiles of PDO-derived cells. Because our findings suggested a spectral continuum rather than separable segments of cell states in dynamic transition between fibroblastic subtypes within FD, organoid cell identities are expected to undergo active conversions. Disambiguating cellular identities in PDOs will require a more rigorous analysis of signature genes within and between datasets. Lastly, discrepancies were observed between the single-cell transcriptional expression patterns and histology. Single-cell transcriptomics were nearly or entirely devoid of osteoprogenitor markers, such as osterix (*SP7*/*OSX)*, *DLX5*, and *ALPL*, as well as mature osteoblast/osteocyte markers, including osteocalcin (*BGLAP*/*OCN*), osteopontin (*SPP1*/*OPN*), and *SOST* (Figure S3). This contradicted histologic findings of positive staining for alkaline phosphatase, osteopontin, and sclerostin in FD tissues [64,86]. Such disparities are expected, as genes at the transcriptome level do not always translate into the proteome, and vice versa [87]. Additionally, the possibility of an altered transcriptome profile during the process of cell extraction and explant culture cannot be ruled out. Considering these potential distortions, PDOs were subjected to various methods of evaluation at the gene, transcript, and protein levels. For future studies, gaining a greater perspective on the molecular aspects of FD is essential for refining the model for better manipulation of environmental and genetic factors, directing the organoids towards the FD phenotype.

## 5. Conclusions

Taken together, our study offers novel insights into FD by unraveling the intralesional cellular and molecular heterogeneity and successfully generating organoids from patient tissues. We illuminate the intricate cellular composition within FD lesions, elucidating the role of fibroblasts, stromal cells, and proliferating populations in driving FD pathogenesis. Furthermore, the ability of PDOs to recapitulate key hallmarks of FD, including mutational status, transcriptional markers, and pathohistological properties associated with the fibrotic events, represents a significant advancement in in vitro modeling of rare bone lesions. While our study is preliminary in nature, these findings highlight the potential of PDO-based models, which, with further refinement, could substantially advance our understanding of underlying pathogenic mechanisms and provide avenues for personalized drug development.

## Figures and Tables

**Table 1:** Donor characteristics and clinical features.

Donor	Gender	Age	Site	Mutation
FD1	M	18	Nasal cavity/maxillary sinus	R201H
FD2	M	19	Zygomaticomaxillary	R201C
FD3	F	25	Hemiface/mandible	R201H
FD4	M	12	Mandible	R201H
FD5	M	14	Forehead/upper orbit	R201H
HV1	F	28	Zygomatic/mandible	None
HV2	F	23	Hemiface/mandible	None
HV3	M	22	Zygomatic/mandible	None
HV4	M	19	Zygomatic/mandible	None
HV5	F	25	Zygomatic/hemiface	None

FD, fibrous dysplasia patient; HV, healthy volunteer.

## Data Availability

The single-cell sequencing data generated for this study have been deposited to the NCBI Gene Expression Omnibus (GEO) (https://www.ncbi.nlm.nih.gov/geo/, accessed on 19 April 2024) under accession number GSE 263294.

## Supplementary Materials

The following supporting information can be downloaded at: https://www.mdpi.com/article/10.3390/cells13090729/s1, Figure S1: Number of genes and UMIs per cell of each sample; Figure S2: Normalized expression of mesenchymal stromal and proliferating canonical cell-type markers; Figure S3: Normalized expression of osteogenic canonical cell-type markers; Table S1: The canonical markers for the eight cell types identified within the FD lesion [40,41,42,43,44,45,46,49,51,52,53,54,55,56,57]; Table S2: Target genes and their primer sequences; Table S3: Cell counts and proportion per sample per cluster; File S1: Top 20 DEGs of clusters.
