# Supplementary material for: Patient-Derived Organoids Recapitulate Pathological Intrinsic and Phenotypic Features of Fibrous Dysplasia
Source: Cells. 2024 Apr 23;13(9):729. doi: 10.3390/cells13090729 (PMC11083396; doi:10.3390/cells13090729)
Supplement: Supplementary file 1 [file cells-13-00729-s001.zip › Patient Consent Form.pdf]

Patient Consent Form for Articles Containing Patient Details and/or Images

This form provides consent for MDPI to publish details and/or images from patients. It must be completed prior to publication.

Patient details

Patient name: ___________________________________

If a representative (e.g. parent, guardian, or next of kin) is signing on the patient's behalf in the event of the patient being underage, deceased or deemed unfit to give legal consent:

Name of patient representative: ___________________________________

Relationship of representative to patient: ___________________________________

By signing this form, I confirm that I have the authority to represent the patient and provide authorization on their behalf.

Article details

Article title: _Patient-Derived Organoids Recapitulate Pathological Intrinsic and Phenotypic Features of Fibrous Dysplasia__

Journal: _____Cells______

Authors: __Ha-Young Kim, Clémentine Charton, Jung Hee Shim, So Young Lim, Jinho Kim, Sejoon Lee, Jung Hun Ohn, Baek Kyu Kim, Chan Yeong Heo___

Declaration by patient or their representative

I, the patient named above or the patient's representative, have read the abovenamed article in full (including text, figures, and supplementary material) and agree to its publication. I am fully aware of the implications of publication and accept any associated risk. In particular, I understand that, despite anonymization, it is possible that I (or the patient) may be identified based on the details or images contained in the article. While the authors and the publisher will make efforts to minimize this risk, confidentiality cannot be guaranteed.

I understand that the paper will be published online in open access format (using a creative commons CC BY 4.0 license, http://creativecommons.org/licenses/by/4.0), meaning that it can be downloaded, copied and reused without limitation. This include any figures, tables, and

supplementary data. The primary audience for the published paper will be healthcare professionals, research academics and students from across the globe.

The final published version may differ from the one submitted to the journal due to minor revisions, changes to style, and reformatting. Publication in the journal mentioned above is not guaranteed and will take place at the discretion of the publisher, and with permission of the Editor-in-Chief (or a qualified Editorial Board member) after a peer review process.

Signing this form does not remove any of my/the patient's statutory rights to privacy. I understand that I may revoke consent at any point prior to publication, but after publication my consent can no longer be withdrawn.

I understand that I/the patient will receive no financial benefit or compensation from publication of the article.

Patient and/or representative signature(s):

____

Place, date:

____
