# Supplementary material for: Patient-Derived Organoids Recapitulate Pathological Intrinsic and Phenotypic Features of Fibrous Dysplasia
Source: Cells. 2024 Apr 23;13(9):729. doi: 10.3390/cells13090729 (PMC11083396; doi:10.3390/cells13090729)
Supplement: Supplementary file 1 [file cells-13-00729-s001.zip › Supplemental Tables.pdf]

Table S1. The canonical markers for the 8 cell types identified within the FD tissue

Cell Type	Canonical Marker Genes	References
Myofibroblast	*ACTA2, TAGLN, TPM2*	[1]
Osteoblast	*SFRP4, SP7, RUNX2, ALPL*	[2,3]
Fibroblast type 1	*KCNMA1, ABI3BP, SMURF2, ADAMTSL1*	[4–7]
Fibroblast type 2	*COL4A1, COL4A2, IGFBP7*	[8]
Proliferating cell	*MKI67, PCNA, TOP2A, CENPF*	[9]
Myogenic cell	*SMYD3, NEAT1, MALAT1, BNC2*	[10–13]
Macrophage	*CD68*	[14]
Dendritic cell	*HLA-DRA, CD74*	[15]

Table S2. Target genes and their primer sequences

Gene Name	Forward primer (5'-3')	Reverse primer (5'-3')
ACTA2	CTATGCCTCTGGACGCACAACT	CAGATCCAGACGCATGATGGCA
TAGLN	TCCAGGTCTGGCTGAAGAATGG	CTGCTCCATCTGCTTGAAGACC
TPM2	GAGAGGTCTGTGGCAAAGTTGG	GGAGGTGATGTCATTGAGTGCG
SFRP4	CTATGACCGTGGCGTGTGCATT	GCTTAGGCGTTTACAGTCAACATC
RUNX2	TGGTTACTGTCATGGCGGGTA	TCTCAGATCGTTGAACCTTGCTA
BICC1	GATGTGTCCCTCCAAGGTTTCC	GGCTTTCGGTAGCACTCAGTTC
DCN	GCTCTCCTACATCCGCATTGCT	GTCCTTTCAGGCTAGCTGCATC
FBLN	GGATACACAGGTGATGGCTTCAC	GTCGCATTCACAGCGGTATCCT
ADAMTSL1	TGACATCGGCTGAGTGCTACGA	GGCATGATCTGCTTGTATCCGTC
KCNMA1	TATCTCTCCAGTGCCTTCGTGG	CTCTCTCGGTTGGCAGACTTGT
SMURF2	TCCTCGGCTGTCTGCTAACTTG	CAGGCATTCTGTGTCATCAGGAC
AB13BP	CCTTCTACACCTAAACGACGCC	GGTGTTGTCCATGTAGGTTCAGG
COL4A1	TGTTGACGGCTTACCTGGAGAC	GGTAGACCAACTCCAGGCTCTC
COL4A2	GGATAACAGGCGTGACTGGAGT	CTTTGCCACCAGGCAGTCCAAT
IGFBP7	GCCATCACCCAGGTCAGCAAG	GGATTCCGATGACCTCACAGCT
MKI67	GAAAGAGTGGCAACCTGCCTTC	GCACCAAGTTTTACTACATCTGCC
PCNA	CAAGTAATGTCGATAAAGAGGAGG	GTGTCACCGTTGAAGAGAGTGG
TOP2	GTGGCAAGGATTCTGCTAGTCC	ACCATTCAGGCTCAACACGCTG
CENPF	AGCACGACTCCAGCTACAAGGT	CATCATGCTTTGGTGTTCTTTCTG
SMYD3	TTACTGCGAGCAGTCCGAGACA	TTGTCCTGGGTTTGGCAACGGA
NEAT1	GCTGGACCTTTCATGTAACGGG	TGAACTCTGCCGGTACAGGGAA
BNC2	ACTCTGCGGGACTATGTCCGAG	ACCGCAGAAACTGCTGAAGGGT
HLA-DRA	AGCTGTGGACAAAGCCAACCTG	CTCTCAGTTCCACAGGGCTGTT
CD74	AAGCCTGTGAGCAAGATGCGCA	AGCAGGTGCATCACATGGTCCT
CD68	CGAGCATCATTCTTTCACCAGCT	ATGAGAGGCAGCAAGATGGACC
MMP9	GCCACTACTGTGCCTTTGAGTC	CCCTCAGAGAATCGCCAGTACT
OSX/SP7	CTGCGGGACTCAACAACTCT	GAGCCATAGGGGTGTGTCAT
DLX5	GAGTAGGTGTCCCGCCTCAGAACCC	CCAACCAGCCAGAGAAAGAA
ALPL	ACCACCACGAGAGTGAACCA	CGTTGTCTGAGTACCAGTCCC
OCN/BGLAP	CACTCCTCGCCCTATTGGC	CCCTCCTGCTTGGACACAAAG
OPN/SPP1	GCCGAGGTGATAGTGTGGTT	GCTTTCCATGTGTGAGGTGAT
DMP1	GATCAGCATCCTGCTCATGTT	AGCCAAATGACCCTTCCATTC
SOST	CCCTTTGAGACCAAAGACGTG	GGCCCATCGGTCACGTAG
RANKL	CCAAGATCTCCAACATGACTTAC	ACCATTAGTTGAAGATACT
CAPG	CTCCATTCCCAGGCTCAGT	GAAACCTCTTCTGGGCCATT
TRAP/ACP5	TTCTACCGCCTGCACTCCAA	AGCTGATCTCCACATAGGCA
IL1β	AAACAGATGAAGTGCTCCTTCCAGG	TGGAGAACACCACTTGTTGCTCCA
TNFα	CTTCTTCTCCTTCCTGATCGTGG	GCTGGTTATCTCTCAGCTCCA
COL1A1	GTGCGATGACGTGATCTGTGA	CGGTGGTTTCTTGGTCGGT
TGFβ1	TCGCCAGAGTGGTTATCTT	TAGTGAACCCGTTGATGTCC
PLOD2	GACAGCGTTCTCTTCGTCCTCA	CTCCAGCCTTTTCGTGGTGACT
GAPDH	ACAGTTGCCATGTAGACC	TTTTTGGTTGAGCACAGG

Table S3. Cell counts and proportion per sample per cluster. The percentage of samples' cells in the cluster as indicated in brackets.

Cluster	FD3	FD4	FD5
Myofibroblast	3,154 (38.1)	3,676 (37.6)	2,055 (17.2)
Osteoblast	2,358 (28.5)	1,969 (20.1)	3,744 (31.3)
Fibroblast type 1	1,358 (16.4)	2,229 (22.8)	3,004 (25.1)
Fibroblast type 2	567 (6.8)	845 (8.6)	1,168 (9.8)
Proliferating S	345 (4.2)	359 (3.7)	831 (6.9)
Proliferating G2M	389 (4.7)	389 (4.0)	602 (5.0)
Myogenic	66 (0.8)	132 (1.4)	362 (3.0)
MPS	51 (0.6)	175 (1.8)	202 (1.7)

Supplementary references

1. Peyser, R.; MacDonnell, S.; Gao, Y.; Cheng, L.; Kim, Y.; Kaplan, T.; Ruan, Q.; Wei, Y.; Ni, M.; Adler, C.; et al. Defining the Activated Fibroblast Population in Lung Fibrosis Using Single-Cell Sequencing. *Am. J. Respir. Cell Mol. Biol.* **2019**, *61*, 74–85.
2. Komori, T. Regulation of Proliferation, Differentiation and Functions of Osteoblasts by Runx2. *Int. J. Mol. Sci.* **2019**, *20*, 1694.
3. Silvent, J.; Nassif, N.; Helary, C.; Azaïs, T.; Sire, J.-Y.; Guille, M.M.G. Collagen Osteoid-Like Model Allows Kinetic Gene Expression Studies of Non-Collagenous Proteins in Relation with Mineral Development to Understand Bone Biomineralization. *PLoS ONE* **2013**, *8*, e57344.
4. Wang, Y.; Guo, Q.; Hei, H.; Tao, J.; Zhou, Y.; Dong, J.; Xin, H.; Cai, H.; Gao, J.; Yu, K.; et al. BK Ablation Attenuates Osteoblast Bone Formation via Integrin Pathway. *Cell Death Dis.* **2019**, *10*, 738.
5. Hodgkinson, C.P.; Naidoo, V.; Patti, K.G.; Gomez, J.A.; Schmeckpeper, J.; Zhang, Z.; Davis, B.; Pratt, R.E.; Mirotsou, M.; Dzau, V.J. Abi3bp Is a Multifunctional Autocrine/Paracrine Factor That Regulates Mesenchymal Stem Cell Biology. *Stem Cells* **2013**, *31*, 1669–1682.
6. Manikoth Ayyathan, D.; Koganti, P.; Marcu-Malina, V.; Litmanovitch, T.; Trakhtenbrot, L.; Emanuelli, A.; Apel-Sarid, L.; Blank, M. SMURF2 Prevents Detrimental Changes to Chromatin, Protecting Human Dermal Fibroblasts from Chromosomal Instability and Tumorigenesis. *Oncogene* **2020**, *39*, 3396–3410.
7. Rypdal, K.B.; Erusappan, P.M.; Melleby, A.O.; Seifert, D.E.; Palmero, S.; Strand, M.E.; Tønnessen, T.; Dahl, C.P.; Almaas, V.; Hubmacher, D.; et al. The Extracellular Matrix Glycoprotein ADAMTSL2 Is Increased in Heart Failure and Inhibits TGFβ Signalling in Cardiac Fibroblasts. *Sci. Rep.* **2021**, *11*, 19757.
8. Kreus, M.; Lehtonen, S.; Skarp, S.; Kaarteenaho, R. Extracellular Matrix Proteins Produced by Stromal Cells in Idiopathic Pulmonary Fibrosis and Lung Adenocarcinoma. *PloS One* **2021**, *16*, e0250109.
9. Li, R.; Wang, T.; Shelp-Peck, E.; Wu, S.-P.; DeMayo, F.J. The Single-Cell Atlas of Cultured Human Endometrial Stromal Cells. *FS Sci.* **2022**, *3*, 349–366.
10. Bobowski-Gerard, M.; Boulet, C.; Zummo, F.P.; Dubois-Chevalier, J.; Gheeraert, C.; Bou Saleh, M.; Strub, J.-M.; Farce, A.; Ploton, M.; Guille, L.; et al. Functional Genomics Uncovers the Transcription Factor BNC2 as Required for Myofibroblastic Activation in Fibrosis. *Nat. Commun.* **2022**, *13*, 5324.
11. Codato, R.; Perichon, M.; Divol, A.; Fung, E.; Sotiropoulos, A.; Bigot, A.; Weitzman, J.B.; Medjkane, S. The SMYD3 Methyltransferase Promotes Myogenesis by Activating the Myogenin Regulatory Network. *Sci. Rep.* **2019**, *9*, 17298.
12. Liu, C.; Gao, X.; Li, Y.; Sun, W.; Xu, Y.; Tan, Y.; Du, R.; Zhong, G.; Zhao, D.; Liu, Z.; et al. The Mechanosensitive lncRNA Neat1 Promotes Osteoblast Function through Paraspeckle-Dependent Smurf1 mRNA Retention. *Bone Res.* **2022**, *10*, 18.
13. Chen, X.; He, L.; Zhao, Y.; Li, Y.; Zhang, S.; Sun, K.; So, K.; Chen, F.; Zhou, L.; Lu, L.; et al. Malat1 Regulates Myogenic Differentiation and Muscle Regeneration through Modulating MyoD Transcriptional Activity. *Cell Discov.* **2017**, *3*, 17002.
14. Bisgaard, L.S.; Mogensen, C.K.; Rosendahl, A.; Cucak, H.; Nielsen, L.B.; Rasmussen, S.E.; Pedersen, T.X. Bone Marrow-Derived and Peritoneal Macrophages Have Different Inflammatory Response to oxLDL and M1/M2 Marker Expression - Implications for Atherosclerosis Research. *Sci. Rep.* **2016**, *6*, 35234.
15. Li, Y.; Jeong, J.; Song, W. Molecular Characteristics and Distribution of Adult Human Corneal Immune Cell Types. *Front. Immunol.* **2022**, *13*, 798346.
