# Supplementary material for: Patient-Derived Organoids Recapitulate Pathological Intrinsic and Phenotypic Features of Fibrous Dysplasia
Source: Cells. 2024 Apr 23;13(9):729. doi: 10.3390/cells13090729 (PMC11083396; doi:10.3390/cells13090729)
Supplement: Supplementary file 1 [file cells-13-00729-s001.zip › V2_Supplemental Figures.pdf]

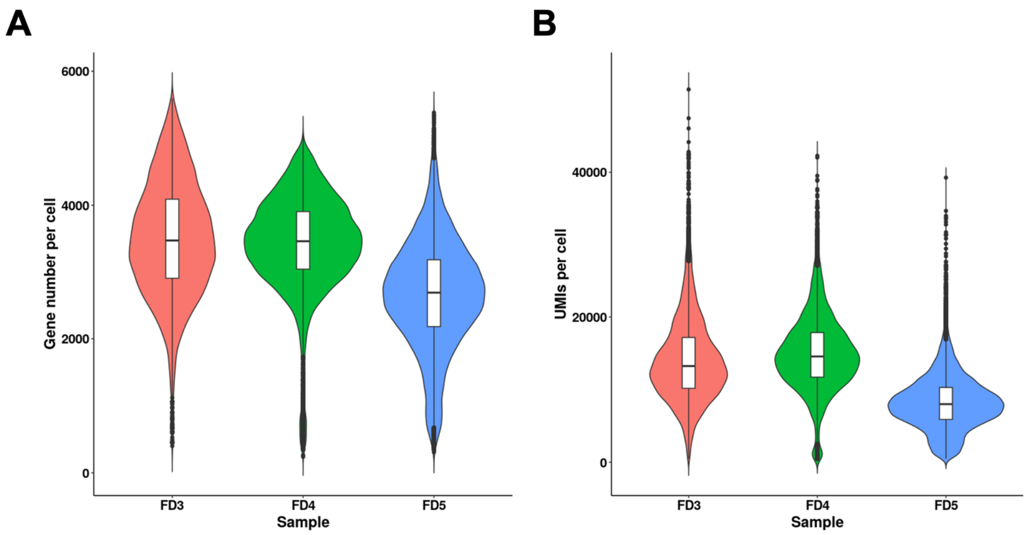

Figure S1. Number of genes and UMIs per cell of each sample. **A,B** Violin plot displaying the distribution of the UMIs per cell and the number of genes per cell in the 3 fibrous dysplasia scRNA-seq samples. The medium point within the boxplots found within each violin plot indicates the median. The lower and upper hinges correspond to the first and third quartiles. The upper whisker extends from the hinge to the largest value no further from the 1.5 × interquartile range (IQR) from the hinge and the lower whiskers extends from the hinger to the smallest value at most 1.5 × from the IQR from the hinge. Outliers are indicated by dots.

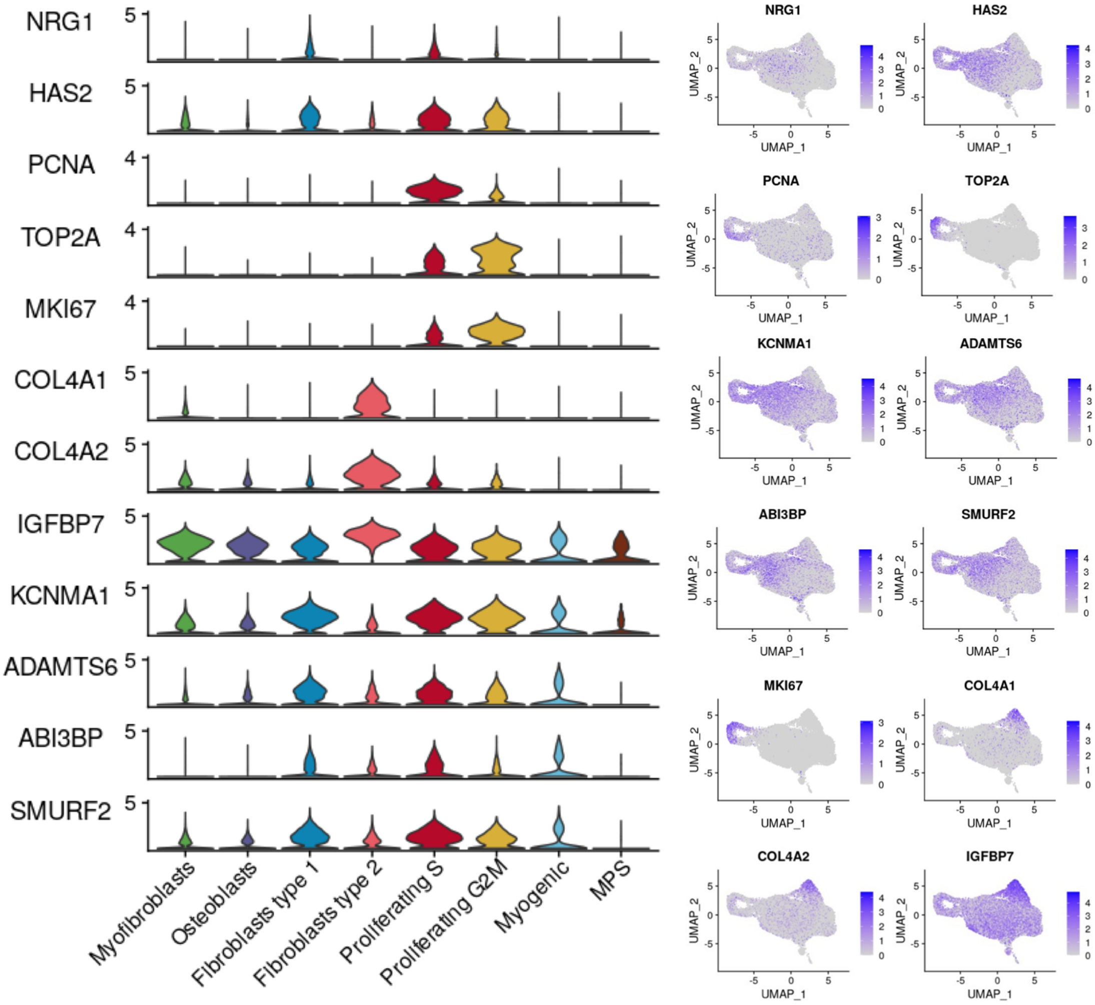

Figure S2. Normalized expression of the mesenchymal stromal and proliferating canonical cell type markers.

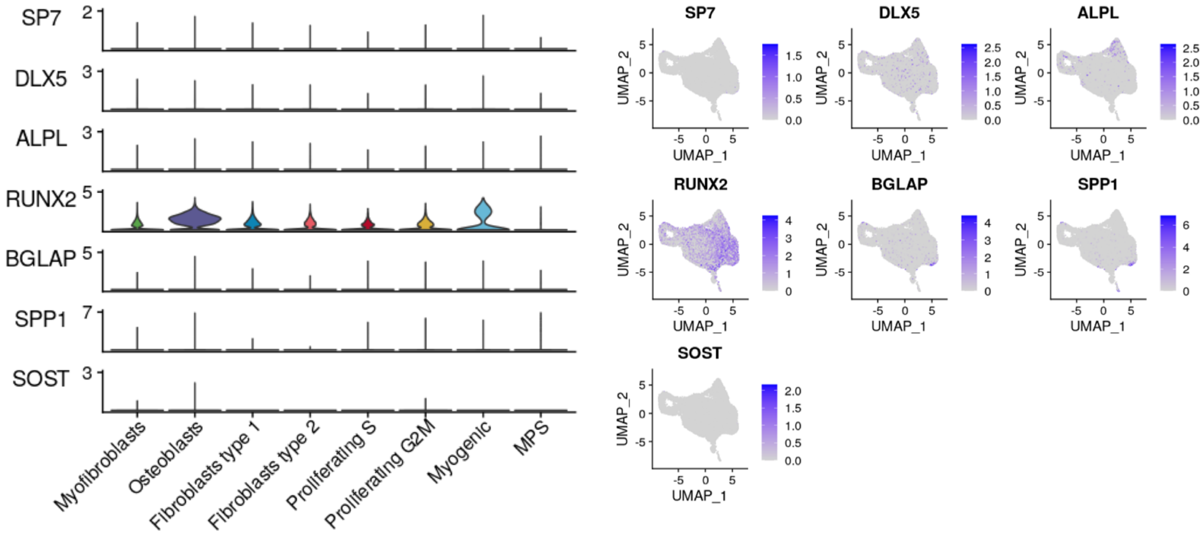

Figure S3. Normalized expression of osteogenic canonical cell type markers.
